# Effect of corruption on perceived difficulties in healthcare access in sub-Saharan Africa

**DOI:** 10.1371/journal.pone.0220583

**Published:** 2019-08-21

**Authors:** Amber Hsiao, Verena Vogt, Wilm Quentin

**Affiliations:** Technische Universität Berlin, Department of Health Care Management, Berlin, Germany; Syracuse University, UNITED STATES

## Abstract

**Background:**

Achieving Universal Health Coverage (UHC) by improving financial protection and effective service coverage is target 3.8 of the Sustainable Development Goals. Little is known, however, about the extent to which paying bribes within healthcare acts as a financial barrier to access and, thus, UHC.

**Methods:**

Using survey data in adults from 32 sub-Saharan African countries in 2014–2015, we constructed a multilevel model to evaluate the relationship between paying bribes and reported difficulties of obtaining medical care. We controlled for individual-, region-, and country-level variables.

**Results:**

Having paid bribes for medical care significantly increased the odds of reporting difficulties in obtaining care by 4.11 (CI: 3.70–4.57) compared to those who never paid bribes, and more than doubled for those who paid bribes often (OR = 9.52; 95% CI: 7.77–11.67). Respondents with higher levels of education and more lived poverty also had increased odds. Those who lived in rural areas or within walking distance to a health clinic had reduced odds of reporting difficulties. Sex, age, living in a capital region, healthcare expenditures per capita, and country Corruption Perception Index were not significant predictors.

**Conclusions:**

We found that bribery in healthcare is a significant barrier to healthcare access, negatively affecting the potential of African countries to make progress toward UHC. Future increases in health expenditures—which are needed in many countries to achieve UHC—should be accompanied by greater efforts to fight corruption in order to avoid wasting money. Measuring and tracking health sector-specific corruption is critical for progress toward UHC.

## Introduction

Universal health coverage (UHC) is recognized by the United Nations (UN) as one of the key strategies for improving global health and wellbeing [1]. As target 3.8 of the Sustainable Development Goals (SDG), global goals set by the UN to improve health and development by 2030, achieving UHC requires that all people have access to quality essential health services without encountering financial barriers or hardships [1]. Although the implementation and financing of UHC varies by country, UN Member States have committed to a broad framework to achieve UHC that includes healthcare financing reform, removing financial risks and barriers to access, and promoting health system efficiency by eliminating waste and corruption [2–4].

In the African region, many countries have made gains in personal healthcare access and quality, which have led to significant improvements in health outcomes. Progress toward UHC has been particularly slow, however, in central and east sub-Saharan African countries [2]. A pan-African survey, the Afrobarometer, found that in 2014–2015, nearly half of Africans forewent needed healthcare, and 4 in 10 of those who accessed care in the prior year found it difficult to access that care [5]. There are several reasons for lack of access to healthcare in Africa. Studies from specific African countries, including Malawi, Nigeria, South Africa, Zambia, and Burkina Faso, have shown that some of these reasons include fear of discrimination or stigmatization [6], lack of education [7], lack of transportation [7–10], and direct financial barriers, such as out-of-pocket payments or user fees [11].

Having to pay illegal fees or bribes to receive medical care is a particular type of financial barrier that has important implications for access to care. This is especially true for poorer patients who are more reliant on public services and thus more vulnerable to bribery [12]. Patients who are frequently confronted with having to pay a bribe at the point of care may decide to delay seeking care until they are much sicker (or may not seek care at all); they may also rely instead on traditional/spiritual healers or informal drug sellers that could exacerbate their health [13]. Of course, patients who decide to pay the bribes are left with fewer resources as well. The repeated action of paying a bribe for medical care may uproot one’s trust in the healthcare system and consequently lead one to perceive that access is limited. While institutional corruption also has consequences for a patient’s access to care, patients do not experience it first-hand as they do with bribery. Public officials may misappropriate or pocket funds, which reduces healthcare system funding overall. Consequently, this leads to fewer resources to purchase necessary medicines, hire qualified healthcare workers, or make improvements to healthcare facilities, which all ultimately impact patient care and quality access. Previous studies have found that perceived national corruption is associated with poorer health across all socioeconomic groups, particularly among the less educated [14]. More generally, poor governance is associated with poorer health outcomes, including lower levels of life expectancy [15], higher mortality rates [15, 16], and lower levels of subjective health feelings [15, 17].

We know that generalized corruption in healthcare has detrimental effects on health in Africa, such as low immunization rates, increased mortality for patients [18], and poor management of chronic conditions [19]. No studies to our knowledge, however, have investigated and quantified the relationship between paying bribes for healthcare—a distinctly different type of corruption in healthcare than institutionalized corruption—and perceived difficulty of access to healthcare across Africa. If the goal is to attain UHC in order to prevent mortality and disability, it is important to understand whether experiencing corruption negatively affects one’s ability or desire to improve health by seeking care [20]. Therefore, our primary study objective is to examine whether paying bribes makes one more likely to report difficulty in obtaining medical care in sub-Saharan Africa. As a second objective, we assess what proportion of these reported difficulties can be explained by individual-level factors, versus region- or country-level factors.

## Methods

We used data from Round 6 of the Afrobarometer survey (2014–2015), a nationally representative publicly available dataset that surveys public attitudes on democracy, governance, economic conditions, and related issues across African countries. Face-to-face interviews are conducted by Afrobarometer staff in the respondent’s language with a randomly selected sample of 1,200 or 2,400 respondents in each country [21]. Only countries in sub-Saharan Africa were included in our analysis, which included 32 countries from Round 6. The Afrobarometer applies a clustered, stratified, multi-stage, area probability sample such that all citizens of voting age have an equal and known chance of selection for interview.

Our outcome of interest was perceived difficulty of obtaining medical treatment. Respondents who had contact with a public clinic or hospital in the past 12 months were asked, “How easy or difficult was it to obtain the medical treatment you needed?” We coded the response as a binary outcome: “easy” (“very easy” and “easy”) and “difficult” (“very difficult” and “difficult”). Our main independent variable of interest was reported frequency of having to pay a bribe for medical treatment. Respondents who had contact with a public clinic or hospital were asked, “How often, if ever, did you have to pay a bribe, give a gift, or do a favor for a health worker or clinic or hospital staff in order to get the medical care you needed? (in the past 12 months)” Possible responses were “never,” “once or twice,” “a few times,” or “often.”

We controlled for the individual-level covariates in our model that have been shown to be associated with access to healthcare. We hypothesized *a priori* that these same covariates might influence an individual’s *perceived* difficulty in obtaining medical treatment. We predicted that proximity to a nearby health clinic (as determined by the interviewer to be within easy walking distance) [22], higher levels of education [14, 23], and being male [24, 25] would be correlated with less perceived difficulty in obtaining medical treatment. In contrast, we predicted that those living in rural areas [23, 26] and those with a higher lived poverty index (LPI) [27, 28] would have more perceived difficulty in obtaining care. The LPI is a measure within the Afrobarometer survey that assesses how frequently the individual surveyed went without basic necessities during the course of the prior year. The index ranges from 0 (no lived poverty) to 5 (extreme lived poverty), and is an average of 5 components that individuals are asked about (frequency of foregoing food, water, medicine, cooking fuel, and cash; the score for each component also ranges from 0 to 5). The LPI is meant to provide a complement to other existing indices of poverty that provides an assessment of the extent to which the interviewee’s basic needs are met [29]. Finally, we hypothesized that those in the younger age groups would have fewer perceived difficulty in obtaining care, though the literature has shown that the type of care sought matters [24, 30]. Missing data was not imputed for our analysis.

We collapsed the number of levels for some of the variables. For education, “some primary schooling” and “primary school completed” were combined, as were “intermediate school or some secondary school/high school” and “secondary school/high school completed.” The four levels of education including and beyond post-secondary qualifications were collapsed into a single category. For urbanity, the Afrobarometer distinguishes between urban, semi-urban, peri-urban, and rural, but only 2 countries (Botswana and Malawi) use the semi-urban and peri-urban values; therefore, we collapsed rural, semi-urban, and peri-urban into a single level “rural” after verifying that the population density of the district was more similar to rural areas of the country as compared with urban areas.

We also controlled for selected region- and country-level variables. A respondent living in the capital may perceive fewer barriers to medical care relative to other regions, since capital regions are often more developed and may have more healthcare resources. Therefore, we accounted for variability in regional development by creating a “capital region” variable. Within each country, the capital region within the Afrobarometer dataset was identified and coded.

At the country level, we merged country-level data from the World Health Organization’s (WHO) Global Health Expenditure database and Transparency International, a global coalition against corruption. From the WHO data, we used healthcare expenditures per capita by country (in constant 2011 international dollars) [31]. From Transparency International, we used the 2015 Corruption Perception Index (CPI) for each country, which is a composite indicator from country experts that measures perception of public sector corruption [32]. We did not find collinearity between CPI and our primary predictor variable (having paid a bribe) (r = –0.13) and thus included both predictors in our models.

We also considered the inclusion of covariates from the WHO related to healthcare access and availability of health services: community health workers per 1,000 people, physicians per 1,000 people, and hospital beds per 1,000 people. Due to sparse and/or outdated data, however, these covariates could not be included in our analysis.

We specified a 3-level multivariable analysis using a random-intercept multilevel logistic regression model. Using a multilevel regression model allowed us to investigate the extent to which there was clustering of outcomes across regions within a country, and between countries. The 3 levels within the model were individuals (n = 29,788), regions within countries (n = 384), and countries (n = 32). With the addition of covariates from each level (i.e., individual-, region-, and country-level), we tested whether the inclusion of the covariates improved our model fit by comparing the Akaike's Information Criterion for each model to the null multilevel logistic regression. We conducted all analyses in Stata/IC 13.1 (College Station, TX).

## Results

In our survey year, 31,322 respondents had contact with a public clinic or hospital (Table 1). Of these, 14% reported paying a bribe or giving a gift at least once in the past year in order to obtain medical care. Our study population was similar in sociodemographic characteristics to the general weighted Afrobarometer population (Table 1).

**Table 1:** Sociodemographic characteristics of study population vs. Afrobarometer total weighted population, 2014–2015*.

	Study Pop. N (%)		Total Pop. N (%)	
**Total**	31,322	(100)	49,137	(100)
**Frequency of paying a bribe or gift**				
Never	26,391	(86)	*— N/A —*	
Once or twice	2,122	(7)		
A few times	1,218	(4)		
Often	805	(3)		
**Sex (%)**				
Male	15,020	(48)	19,126	(50)
Female	16,302	(52)	19,275	(50)
**Age group (%)**				
18–25 yrs	7,242	(23)	9,352	(24)
26–35 yrs	9,769	(31)	11,534	(30)
36–45 yrs	6,533	(21)	7,682	(20)
46–55 yrs	3,838	(12)	4,793	(13)
>55 yrs	3,772	(12)	4,847	(13)
**Education (%)**				
No formal schooling	4,092	(13)	5,886	(15)
Informal schooling only	1,473	(5)	2,110	(6)
Some or primary schooling completed	9,531	(30)	10,680	(28)
Intermediate or some secondary/high	6,620	(21)	8,534	(22)
Secondary/high school completed	5,060	(16)	5,503	(14)
Post-secondary or higher	4,480	(14)	5,597	(15)
**Urban/rural**				
Urban	11,693	(37)	14,903	(39)
Rural	19,629	(63)	23,499	(61)
**Health clinic access in EA or PSU**				
No	12,582	(41)	14,942	(39)
Yes	18,461	(59)	23,170	(61)
**Lived poverty index**				
No lived poverty	4,680	(15)	6,292	(17)
>0 to ≤1	10,726	(35)	12,540	(33)
>1 to ≤2	10,058	(32)	12,079	(32)
>2 to ≤3	4,771	(15)	6,057	(16)
>3 to ≤4	830	(3)	1,090	(3)

* Sum of counts for each characteristic may not add up to column total due to missing data.

**Fig 1:**
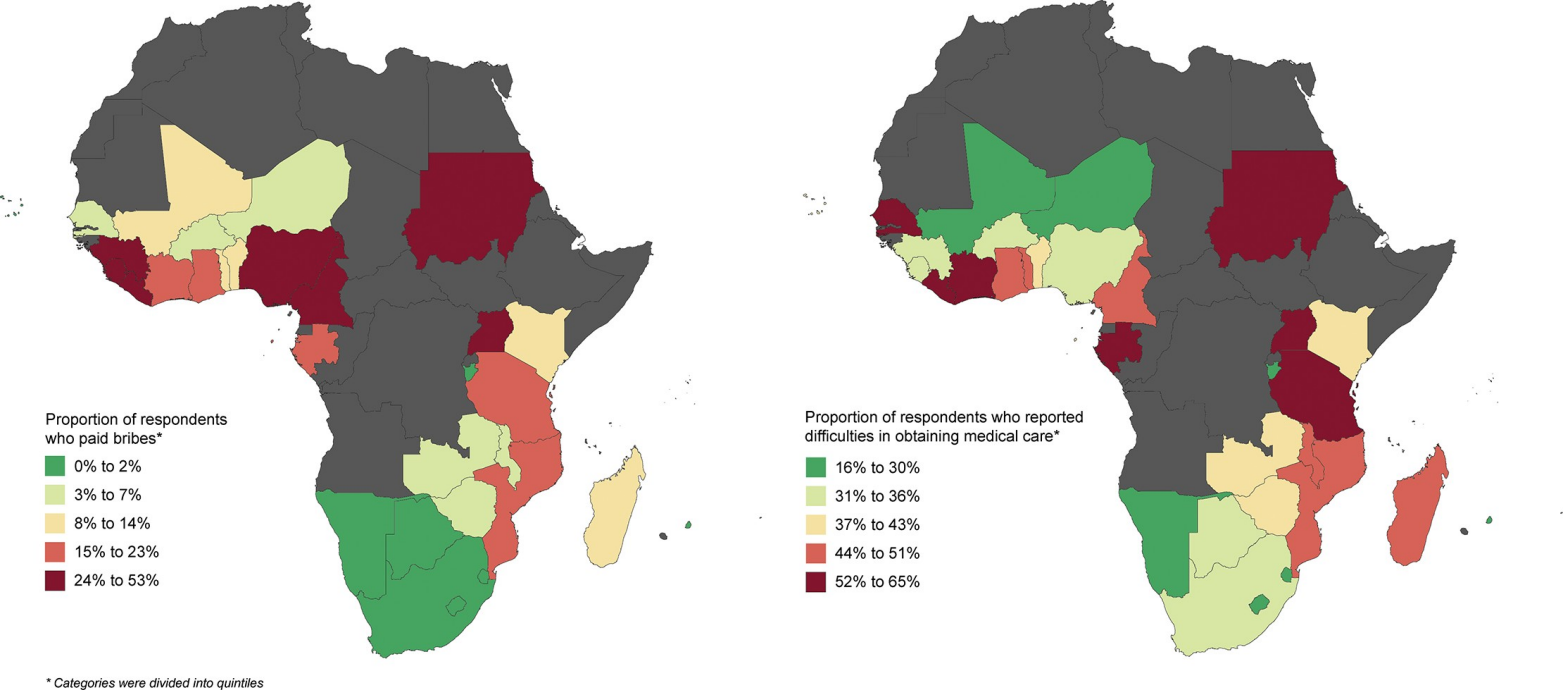
Proportion of respondents who paid bribes versus reported difficulties in obtaining care in sub-Saharan Africa, 2014–2015.

Forty-one percent of respondents (n = 12,821) said it was difficult to obtain medical care (**Table 1**). By country, the highest proportions of respondents that reported it being difficult to obtain medical care were in Gabon (65%), Liberia (63%), Sudan (62%), and Senegal (60%). The highest proportions of respondents that reported ever paying bribes were in Liberia (53%), Sudan (32%), Cameroon (30%), Guinea (25%), and Sierra Leone (25%) (Fig 1 and S1 Table**)**.

In Model 1, where we only included the main predictor (frequency of paying bribes), we found that those who paid bribes once or twice had 4.11 (95% confidence interval [CI]: 3.70–4.57) times the odds of reporting that it was difficult to obtain medical care compared with those who never paid bribes (Table 2). The odds further increased for those who paid bribes a few times (OR = 4.90; 95% CI: 4.25–5.65) and more than doubled for those who paid bribes often (OR = 9.52; 95% CI: 7.77–11.67).

**Table 2:** Odds ratio and 95% confidence intervals for associations between having paid a bribe to obtain medical care and perceived difficulty of obtaining medical care.

	Model 1		Model 2		Model 3		Model 4	
	OR	95% CI	OR	95% CI	OR	95% CI	OR	95% CI
**Paid a bribe to obtain medical care**								
Never	1	Reference	1	Reference	1	Reference	1	Reference
Once or twice	*4.11	(3.70–4.57)	*4.00	(3.59–4.46)	*4.00	(3.59–4.46)	*3.99	(3.58–4.45)
A few times	*4.90	(4.25–5.65)	*4.73	(4.08–5.47)	*4.72	(4.08–5.47)	*4.69	(4.05–5.43)
Often	*9.52	(7.77–11.67)	*9.12	(7.36–11.30)	*9.11	(7.35–11.29)	*9.07	(7.32–11.24)
**Health clinic access within walking distance**								
No			1	Reference	1	Reference	1	Reference
Yes			*0.86	(0.81–0.91)	*0.86	(0.81–0.91)	*0.87	(0.81–0.92)
**Urbanity**								
Urban			1	Reference	1	Reference	1	Reference
Rural			*0.85	(0.79–0.91)	*0.85	(0.80–0.91)	*0.86	(0.80–0.92)
**Lived poverty index**								
No lived poverty			1	Reference	1	Reference	1	Reference
>0 to ≤1			*1.38	(1.26–1.50)	*1.38	(1.26–1.51)	*1.41	(1.28–1.54)
>1 to ≤2			*2.18	(1.98–2.39)	*2.18	(1.98–2.39)	*2.22	(2.02–2.45)
>2 to ≤3			*2.97	(2.66–3.31)	*2.97	(2.66–3.31)	*3.06	(2.74–3.41)
>3 to ≤4			*4.06	(3.37–4.89)	*4.06	(3.37–4.89)	*4.20	(3.48–5.07)
**Education**								
No formal schooling			1	Reference	1	Reference	1	Reference
Informal schooling only (including Koranic)			1.01	(0.87–1.17)	1.01	(0.87–1.17)	1.01	(0.87–1.18)
Some or primary schooling completed			1.02	(0.93–1.12)	1.02	(0.93–1.12)	1.03	(0.93–1.13)
Intermediate school or some secondary/high school			*1.16	(1.05–1.28)	*1.16	(1.04–1.28)	*1.17	(1.06–1.30)
Secondary/high school completed			*1.20	(1.08–1.34)	*1.20	(1.07–1.34)	*1.20	(1.07–1.34)
Post-secondary or higher			*1.14	(1.02–1.28)	*1.14	(1.02–1.28)	*1.15	(1.02–1.29)
**Sex**								
Male			1	Reference	1	Reference	1	Reference
Female			0.95	(0.90–1.00)	0.95	(0.90–1.00)	0.96	(0.91–1.01)
**Age group**								
18–25 yrs			1	Reference	1	Reference	1	Reference
26–35 yrs			1.08	(1.00–1.16)	1.08	(1.00–1.16)	1.07	(0.99–1.15)
36–45 yrs			1.05	(0.97–1.14)	1.05	(0.97–1.14)	1.05	(0.97–1.14)
46–55 yrs			1.00	(0.91–1.09)	1.00	(0.91–1.09)	0.99	(0.90–1.09)
>55 yrs			0.93	(0.84–1.03)	0.93	(0.84–1.03)	0.93	(0.84–1.02)
**Capital region**								
Yes					1	Reference	1	Reference
No					1.22	(0.99–1.51)	1.24	(1.00–1.54)
**Corruption Perceptions Index (CPI)**								
							1.00	(0.98–1.02)
**Healthcare expenditures per capita**								
							1.00	(1.00–1.00)
*ICC (SE) for country*	0.07 (0.02)		0.05 (0.02)		0.06 (0.02)		0.05 (0.02)	
*ICC (SE) for region*	0.14 (0.02)		0.13 (0.02)		0.13 (0.02)		0.13 (0.02)	
*Akaike's information criterion (AIC)*, *% change*	36,893.04, –6.8%		35,325.45, –10.8%		35,323.99, –10.8%		34,526.47, –12.8%	

**Notes:** The ICC (SE) in the null model for country was 0.09 (0.02) and for region 0.16 (0.02). The AIC for the null model was 39,595.94. The proportional change in AIC compares each iteration of the model with the null (e.g, Model 1 compared to null, Model 2 compared to Model 1).

*Statistically significant at ⍺ = 0.05

In Model 2, individual-level covariates were added to our multilevel Model 1. We found that the odds of reporting that it was difficult to obtain medical care did not substantially change when compared to the null model (Model 1). Respondents with at least some secondary education (OR = 1.16–1.20) or who had a higher LPI (OR = 1.38–4.06) had increased odds of reporting difficulties in obtaining care. We observed that respondents with geographic access to a health clinic (OR = 0.86; 95% CI: 0.81–0.91) and those living in a rural area (OR = 0.85; 95% CI: 0.79–0.91) had reduced odds of reporting difficulties in obtaining care.

In Model 3, we added the capital region covariate, which was not found to be significant. A sensitivity analysis was also conducted by including “secondary” cities (e.g., Johannesburg, South Africa) in the coding of the capital region covariate, but there was no effect on the analysis. In Model 4, our full model with the country-level covariates added, both CPI and health expenditures per capita were not significant. The ORs (and associated p-values) for our primary exposure of interest (paying a bribe) in all of the models remained stable with each iteration of the model.

We found that the intraclass correlation coefficient in Model 4 was 0.05 for the between-country variation and 0.13 for the between-region variation, indicating that approximately 5% and 13% of the total variance in the outcome was due to unexplained between-country and between-region variation in reporting difficulties in obtaining care, respectively.

We also plotted the estimated country- and region-level residuals to examine the country- and region-level effects (S1 and S2 Figs). The figures show that for approximately 13 countries and a substantial number of regions, the perceived difficulty of obtaining care is significantly above or below the average values.

## Discussion

To our knowledge, our study is the first to explore whether individually perceived corruption in the form of bribes might impact one’s perception of how difficult it is to obtain medical care in the African region. We found that patients who had paid bribes had between 4.00–9.11 times the odds of reporting difficulties in obtaining medical care, compared to those who never paid bribes. Only poverty was associated with a similarly large increase in the odds of reporting difficulties in obtaining medical care (OR 1.41–4.20, depending on the level of poverty). Country level factors, such as health expenditures per capita and corruption as measured by the CPI were not important. Our findings have important implications for policymakers and researchers in the context of the current quest of African countries to reach UHC.

First, we find a high incidence of patients having to pay bribes for medical care in some African countries, reaching as high as 53% in Liberia, and incidence is above 10% in half of the countries. This is a reason for concern, as the pervasiveness of paying bribes makes it difficult for many patients to access care. Therefore, fighting health sector corruption should be an integral part of current efforts to reach UHC [20]. As corruption is often deeply-engrained in societies, this will likely require substantial changes to the health system in addition to changes to policies that reach beyond the health sector [33]. Studies have highlighted that fighting corruption in the health sector must start from the top: Ministers of Health must demonstrate strong leadership, and high morality and integrity in order to change the attitudes of providers and patients who may have accepted bribery as a cultural trait of the health system [19]. In addition, moving away from direct payments can reduce corruption because it eliminates the exchange of money at the point of access to care [4]. Furthermore, more transparency between patients and healthcare providers is needed; for example, information on user charges and exemptions should be clear and easily accessible to patients. Fighting corruption also requires strong institutions that are willing to proactively collect patient complaints, investigate corruption allegations, and punish corruption [19, 34].

On the patient side, there is evidence that beyond the healthcare system itself, the social networks of the patients may play a role in perpetuating corruption. A recent study found that in Tanzania and Uganda, those who reported strong social networks perceived fewer barriers to health access and were less prone to extortive bribing. If patients know that health access is challenging, they may mobilize their existing social networks for financial assistance and/or direct connections to health workers for favors and prioritized access [34]. In contrast, in Rwanda, authorities used a “naming and shaming” approach to actively publish the names of those engaged in crimes of corruption (along with the names of their parents and their communities of origin). This aggressive approach has reduced reliance on social networks for favors. These findings suggest that fighting corruption has local and country-specific nuances that must be understood before implementing policies, but also that social networks beyond the health system itself are potential a point of intervention for battling corruption.

Second, there has been significant international attention on health system financing to achieve UHC [35, 36]. The WHO has emphasized that low-income countries will require additional financial support in order to achieve UHC and expand access to services [4]. However, we found that healthcare expenditures per capita had no significant impact on the odds of reporting difficulties in obtaining medical care, and this finding did not change in our sensitivity analyses, when we used public expenditures per capita or the log of healthcare expenditures. This suggests that there may be inefficiencies in how available healthcare resources in sub-Saharan Africa are currently being used to improve patients’ access to care, though there is likely significant variation within and between countries that our current study does not attempt to address. Some of this variation is likely due to the presence of social networks described above that may pervert equitable access, especially for the poorest [34]. At the same time, it may also suggest that more resources will not lead to better outcomes unless efforts are increased to fight corruption in the healthcare sector [19].

Third, when we compare our data on reported difficulties in obtaining medical care with the UHC service coverage index, which is used by the UN to monitor progress toward SDG 3.8.1 [3], we find that countries that have a low UHC index score (e.g., Liberia has a UHC index score of 34/100) also tend to have a higher proportion of respondents saying it is difficult to obtain medical care (63% in Liberia), but the correlation between the two is relatively low. This may indicate that the UHC coverage index is not a good reflection of the experiences of patients living in African countries: Niger has a low UHC index score of 33, but also has one of the lowest proportions (23%) of respondents saying that they had difficulty obtaining medical care. Conversely, Gabon has a UHC index score of 52, but 65% of respondents (the highest in our study population) say it was difficult to obtain medical care. The UHC index is based on information that has generally been available in most countries, including vaccination coverage and health worker density; however, various standalone components in the index for a specific country are often outdated (i.e., more than 10 years old) and recency of primary data availability impacts a country’s UHC score. Further, since the index does not include a component that measures actual patient access or patient satisfaction, this lack of correlation is not entirely surprising. Our findings suggest that survey data concerning difficulty of obtaining medical care may provide additional insights on effective service coverage that could be used to supplement existing metrics as part of the SDG monitoring process. Furthermore, given the strong influence of bribes on difficulty in obtaining medical care, monitoring country-level healthcare corruption levels is important for achieving UHC because fighting corruption is possible only if change is measured [33]. Many African countries implement Demographic and Health Surveys that routinely monitor the impact of various indicators on health; a bribery question similar to the Afrobarometer question could be added to country surveys to routinely track healthcare corruption over time.

Fourth, our analysis confirms some existing knowledge and raises several questions that require further analysis. The findings from our study confirm that the poor have more difficulty obtaining care—up to 4.20 times more—which is in line with existing literature that the impoverished experience more financial hardships in accessing care [1]. Similarly, geographic proximity to a health clinic makes it easier to obtain care (OR = 0.87; 95% CI: 0.81–0.92). However, interestingly and against our expectation, living in a rural area also reduces the odds of reporting difficulties in obtaining care (OR = 0.86; 95% CI: 0.80–0.92). This could be related to greater social inequalities in urban areas [37], and a greater general awareness of the difficulties in obtaining care in an urban setting. For example, urban areas may attract more experienced health professionals who are more in demand, and poorer individuals may feel especially disadvantaged when they seek care due to finances or discrimination. Another possible explanation could be that the (in)existence of geographic access problems is captured by the covariate “proximity to a nearby clinic,” whereas the “rural” covariate only captures factors unrelated to physical access. One study with similar findings has speculated that rural populations have lower expectations of access to health services [38]; therefore, the “rural” covariate may address these lower expectations because geographic access is already captured in the “proximity to a nearby clinic” covariate. Furthermore, the more educated reported more difficulties in obtaining care. We theorize that one possible explanation for this is that those who are more educated may be better informed about their rights and may demand more from the healthcare system [39]. Finally, national level corruption as measured by the CPI was not found to be significant. This might indicate that while the CPI may be an appropriate measure of broader institutionalized corruption, it may not necessarily capture corruption in the form of bribery as experienced by patients accessing care. Further, because the CPI relies on a range of data sources that reflect expert opinions, it may differ from the experiences of the general population [20, 33, 40].

Our study has limitations. The Afrobarometer survey asked only those who received medical care in the prior year about difficulties of obtaining medical care. Thus, our results may underestimate the effect of paying bribes on difficulties in obtaining care because our analysis does not include those who attempted to obtain care, but never received it. Furthermore, the pervasiveness of bribes within a healthcare system may normalize the behavior so much that it may be difficult for patients to distinguish which payments are legal versus illegal (i.e., underreporting of bribes paid, confusing it with user fees). Our analysis also excludes respondents who may have avoided the medical system altogether even when it was necessary. US-based studies suggest that individuals may avoid medical care when there are barriers to access—even when they are insured [41], and the same is likely to be true in African setting. Distrust in the medical profession may also decrease one’s willingness to seek care [42]. Finally, our study does not allow us to identify the reasons for the considerable variation across countries. More research is needed to understand corruption within the context of each individual country’s health policies and systems, and to identify possible actions to prevent bribery.

In summary, our study found that bribery is strongly associated with increased difficulties in obtaining medical care in sub-Saharan Africa. Therefore, national governments and international actors should increase their efforts to fight corruption and to measure it in order to make progress toward achieving UHC. Given our finding that higher health expenditures are not associated with easier access to medical care, future increases in health expenditures should be accompanied by even greater efforts to fight corruption to avoid wasting money. Finally, monitoring progress toward UHC would benefit from including survey data on the incidence of corruption and on perceived difficulty in obtaining medical care.

## Supporting information

S1 Table Difficulty of obtaining medical care by country, 2014–2015 (n = 31,322). (DOCX)

S1 Fig Country-level residuals for reported difficulty of obtaining care. (PNG)

S2 Fig Region-level residuals for reported difficulty of obtaining care. (PNG)
