# Correction: Effect of corruption on perceived difficulties in healthcare access in sub-Saharan Africa

**DOI:** 10.1371/journal.pone.0224915

**Published:** 2019-11-01

**Authors:** Amber Hsiao, Verena Vogt, Wilm Quentin

The Funding statement is incorrect. The correct Funding statement is as follows: We acknowledge support by the German Research Foundation and the Open Access Publication Fund of TU Berlin.
