# Supplementary material for: Effect of corruption on perceived difficulties in healthcare access in sub-Saharan Africa
Source: PLoS One. 2019 Aug 21;14(8):e0220583. doi: 10.1371/journal.pone.0220583 (PMC6703670; doi:10.1371/journal.pone.0220583)
Supplement: S1 Table — (DOCX) [file pone.0220583.s001.docx]

**S1 Table. Difficulty of obtaining medical care by country, 2014-2015 (n=31,322)**

| **Country** | **Difficulty of obtaining medical care (N=31,322)** | | | | | **Paid bribes to obtain medical care (N=30,536)** | | | |
| --- | --- | --- | --- | --- | --- | --- | --- | --- | --- |
|  | **Easy**  **n (%)** | | **Difficult**  **n (%)** | | | **Never**  **n (%)** | | **Ever**  **n (%)** | |
| Benin | 393 | (57) | 291 | (43) |  | 595 | (87) | 86 | (13) |
| Botswana | 654 | (67) | 319 | (33) |  | 973 | (100) | 0 | (0) |
| Burkina Faso | 473 | (64) | 263 | (36) |  | 710 | (96) | 26 | (4) |
| Burundi | 652 | (81) | 153 | (19) |  | 784 | (98) | 19 | (2) |
| Cameroon | 387 | (48) | 411 | (52) |  | 532 | (70) | 227 | (30) |
| Cape Verde | 445 | (59) | 313 | (41) |  | 747 | (99) | 9 | (1) |
| Cote d'Ivoire | 254 | (46) | 303 | (54) |  | 452 | (82) | 100 | (18) |
| Gabon | 241 | (35) | 444 | (65) |  | 545 | (80) | 140 | (20) |
| Ghana | 571 | (55) | 459 | (45) |  | 771 | (83) | 162 | (17) |
| Guinea | 413 | (64) | 229 | (36) |  | 477 | (75) | 158 | (25) |
| Kenya | 1,021 | (57) | 761 | (43) |  | 1,533 | (88) | 202 | (12) |
| Lesotho | 456 | (71) | 187 | (29) |  | 623 | (98) | 14 | (2) |
| Liberia | 325 | (37) | 548 | (63) |  | 391 | (47) | 444 | (53) |
| Madagascar | 259 | (56) | 200 | (44) |  | 415 | (92) | 38 | (8) |
| Malawi | 885 | (53) | 775 | (47) |  | 1,497 | (94) | 90 | (6) |
| Mali | 544 | (75) | 180 | (25) |  | 660 | (92) | 58 | (8) |
| Mauritius | 769 | (84) | 146 | (16) |  | 914 | (100) | 1 | (0.1) |
| Mozambique | 912 | (53) | 801 | (47) |  | 1,315 | (77) | 387 | (23) |
| Namibia | 693 | (75) | 228 | (25) |  | 884 | (98) | 20 | (2) |
| Niger | 606 | (77) | 178 | (23) |  | 725 | (95) | 37 | (5) |
| Nigeria | 1,093 | (67) | 545 | (33) |  | 1,204 | (76) | 390 | (24) |
| Sao Tome and Principe | 488 | (63) | 282 | (37) |  | 590 | (83) | 120 | (17) |
| Senegal | 288 | (40) | 431 | (60) |  | 692 | (97) | 22 | (3) |
| Sierra Leone | 449 | (68) | 216 | (32) |  | 479 | (75) | 160 | (25) |
| South Africa | 957 | (68) | 455 | (32) |  | 1,198 | (97) | 31 | (3) |
| Sudan | 306 | (38) | 493 | (62) |  | 532 | (68) | 253 | (32) |
| Swaziland | 622 | (79) | 166 | (21) |  | 775 | (98) | 12 | (2) |
| Tanzania | 784 | (45) | 947 | (55) |  | 1,388 | (81) | 327 | (19) |
| Togo | 267 | (48) | 287 | (52) |  | 493 | (89) | 61 | (11) |
| Uganda | 863 | (48) | 936 | (52) |  | 1,362 | (76) | 437 | (24) |
| Zambia | 524 | (61) | 332 | (39) |  | 776 | (95) | 42 | (5) |
| Zimbabwe | 907 | (63) | 542 | (37) |  | 1,359 | (95) | 72 | (5) |
| **Total** | **18,501** | **(59)** | **12,821** | **(41)** |  | **26,391** | **(86)** | **4,145** | **(14)** |
