# Supplementary figures and images for: Effect of corruption on perceived difficulties in healthcare access in sub-Saharan Africa
Source: PLoS One. 2019 Aug 21;14(8):e0220583. doi: 10.1371/journal.pone.0220583 (PMC6703670; doi:10.1371/journal.pone.0220583)

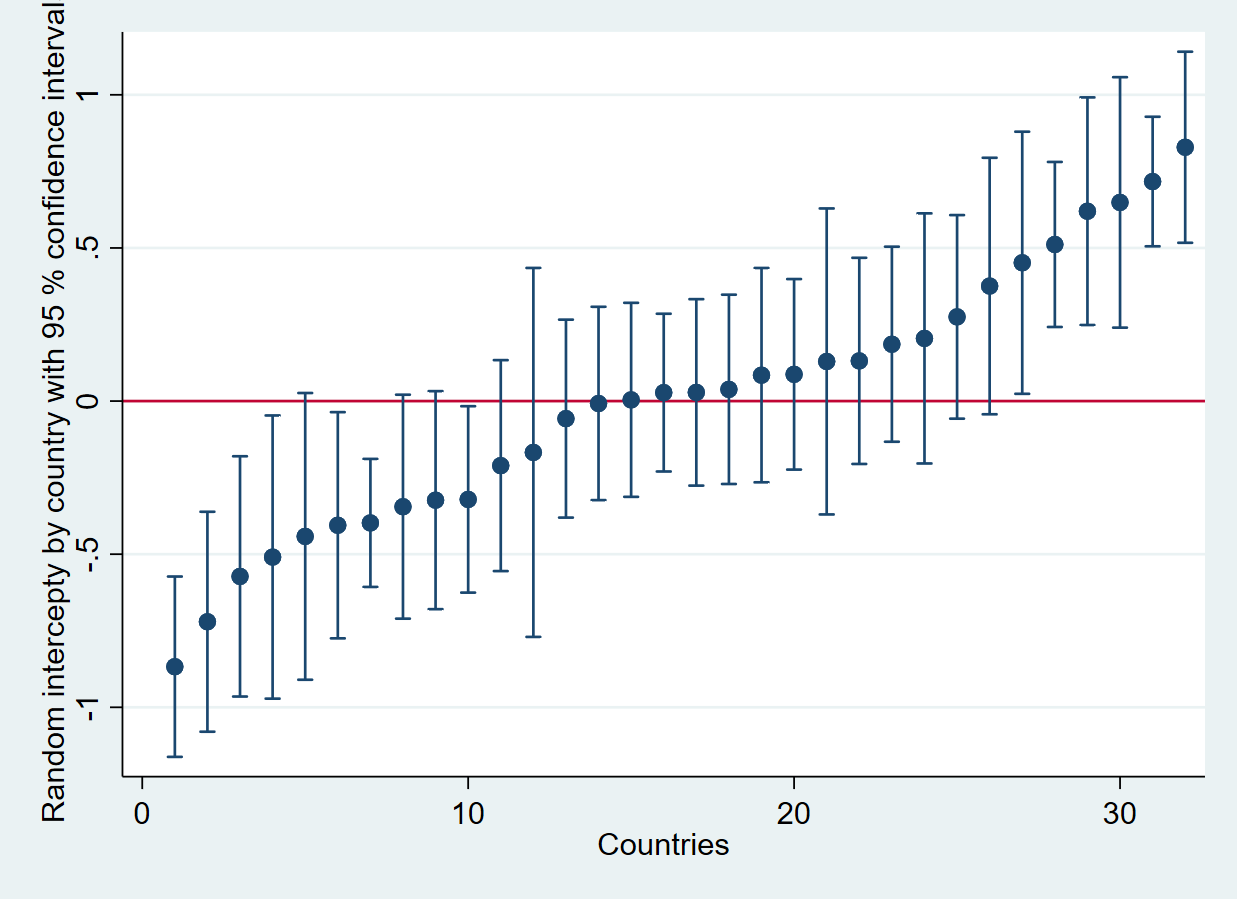

Supplement: S1 Fig — (PNG) [file pone.0220583.s002.png]

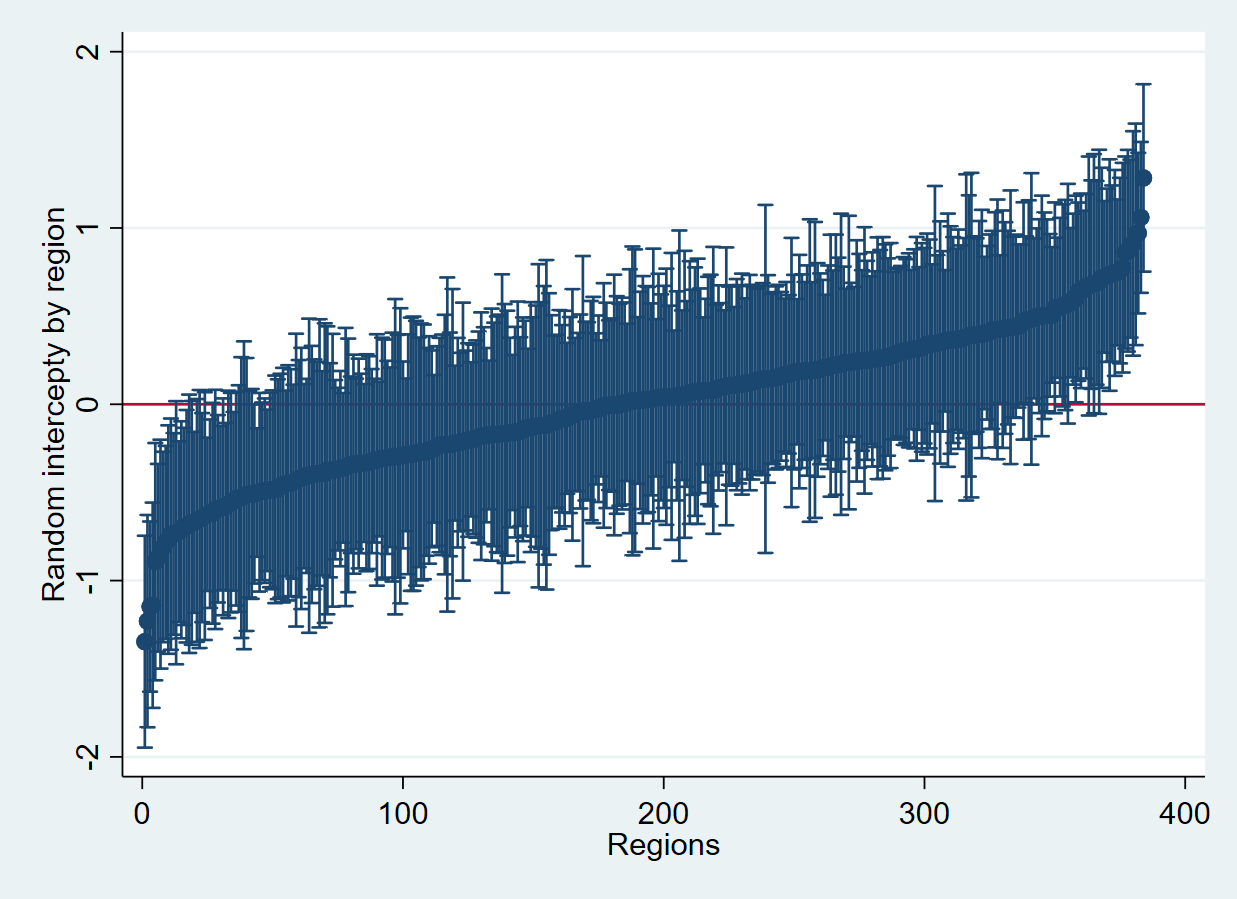

Supplement: S2 Fig — (PNG) [file pone.0220583.s003.png]
